# Benzodiazepine and Z-drug use and risk of pneumonia in patients with chronic kidney disease: A population-based nested case-control study

**DOI:** 10.1371/journal.pone.0179472

**Published:** 2017-07-10

**Authors:** Meng-Ting Wang, Yun-Han Wang, Hsin-An Chang, Chen-Liang Tsai, Ya-Sung Yang, Chen Wei Lin, Cheng-Chin Kuo, Yu-Juei Hsu

**Affiliations:** 1 School of Pharmacy, National Defense Medical Center, Taipei, Taiwan, Republic of China; 2 Department of Psychiatry, Tri-Service General Hospital, National Defense Medical Center, Taipei, Taiwan, Republic of China; 3 Division of Pulmonary and Critical Care, Department of Internal Medicine, Tri-Service General Hospital, National Defense Medical Center, Taipei, Taiwan, Republic of China; 4 Division of Infectious Diseases and Tropical Medicine, Department of Medicine, Tri-Service General Hospital, National Defense Medical Center, Taipei, Taiwan, Republic of China; 5 Institute of Cellular and System Medicine, National Health Research Institutes, Zhunan, Taiwan, Republic of China; 6 Graduate Institutes of Life Sciences and Biochemistry, National Defense Medical Center, Taipei, Taiwan, Republic of China; 7 Metabolomic Research Center, China Medical University Hospital and Graduate Institute of Basic Medical Science, China Medical University, Taichung, Taiwan, Republic of China; 8 Division of Nephrology, Department of Medicine, Tri-Service General Hospital, National Defense Medical Center, Taipei, Taiwan, Republic of China; 9 Graduate Institutes of Medical Sciences and Biochemistry, National Defense Medical Center, Taipei, Taiwan, Republic of China; Kaohsiung Medical University Hospital, TAIWAN

## Abstract

**Background:**

Concerns were raised about pneumonia development from benzodiazepines (BZDs) and Z-drugs, but direct evidence is limited, conflicting and without examining the highly susceptible patients with chronic kidney disease (CKD) nor specifying the risk for different drug utilizations. This study aimed to investigate whether use of BZDs and Z-drugs was each associated with an increased risk of pneumonia in a CKD population.

**Methods:**

We performed a nested case-control study of 36,880 CKD patients analyzing the Taiwan National Health Insurance Database between 01/1/2000 and 12/31/2011. Among the study cohort, we identified 4,533 cases of pneumonia based on validated disease codes, chest x-ray examination, and prescriptions of respiratory antibiotics, and randomly selected 16,388 controls from risk sets, matched by sex, age, and number of CKD-related hospitalizations. All prescription filling records of BZDs and Z-drugs in the year before the event/index date were analyzed for cases and controls. Conditional logistic regressions were performed to estimate the odds ratios (ORs).

**Results:**

Current use of BZDs was associated with a 1.31-fold (95% CI, 1.18–1.26) increased risk of pneumonia compared to nonuse, but not for recent and past use. The risk from current BZD use was confined to new initiation (adjusted OR, 2.47; 95% CI, 2.02–3.03) or use for ≤ 30 days, and elevated to 2.88-fold (95% CI, 1.87–4.42) with parenteral administration. New initiation and current short-term use of Z-drugs was associated with a 2.94-fold (95% CI, 1.65–5.26) and 1.75-fold (95% CI, 1.13–2.72) increased risk of pneumonia, respectively. The findings were robust to adoption of a case-crossover study that analyzed cases only.

**Conclusions:**

Use of BZRAs is associated with an increased risk of pneumonia in CKD patients, especially for patients newly initiating BZDs or Z-drugs or those injected with BZDs. Physicians should exercise cautions for signs of pneumonia when prescribing BZDs or Z-drugs to CKD patients.

## Introduction

Benzodiazepine receptor agonists (BZRAs), including benzodiazepines (BZDs) and Z-drugs, are the mainstay treatments for insomnia and anxiety [1] and the most commonly prescribed psychotropic medications in many countries [2]. In the United States, the annual number of BZRA prescriptions for insomnia continues to rise, exceeding 18 million outpatient prescriptions in 2009 [3]. Accordingly, examination of BZRA’s safety profile is clinically critical. Studies have typically focused on BZRA’s effect on central nervous system such as fall-related fractures [4], and impaired cognitive function [5,6]. Recently, animal studies have shown that BZRAs could be a risk factor for pneumonia probably through direct suppression of innate immunity [7]. Notably, pneumonia is a top cause of hospitalization that results in approximately 50,000 deaths annually (American Thoracic Society, 2015) [8], and leads to a significant mortality rate as high as 23% within 30 days after disease occurrence [9], which makes such preventable drug-induced pneumonia a primary health management target.

Pneumonia is a highly fatal infectious disease and has been recently linked to the use of BZRAs [10–13]. However, only two population-based studies had primarily investigated the association between pneumonia and BZRAs, but yielded opposite findings [14,15]. Obiora et al [14] found an increased risk of pneumonia from current, recent, and remote use of BZRAs. In contrast, Dublin et al [15] showed that current use of benzodiazepines did not associate with pneumonia in an elderly population, and past use slightly reduced the risk of pneumonia. Nonetheless, the two studies were mainly limited by imprecise measurement of BZRA use [14,15], uncertainty of pneumonia diagnosis [14], and small sample sizes [15] as well as recall bias [15]. Accordingly, this drug safety concern requires further elucidation.

Moreover, the association between BZRAs and pneumonia has not been specifically characterized in the infection-prone patients with chronic kidney disease (CKD). The CKD patients have a more than two-fold higher risk of pneumonia than general population [16], and a substantial proportion of them encounter insomnia [17] or anxiety [18], which makes them more likely to use BZRAs. In addition, pneumonia is a substantial cause of morbidity and mortality in CKD patients [19], with up to a 4.9-fold increased death rate compared to a non-CKD population [16].

This study aimed to evaluate the separate impacts of BZDs and Z-drugs on the risk of pneumonia in a CKD population by different timing of drug usages, and to investigate if the association varies depending on dose, duration, route of administration, and individual agents of BZDs and Z-drugs.

## Materials and methods

### Study design and data source

A population-based nested case-control study was performed by analyzing the Taiwan Longitudinal Health Insurance Database (LHID) spanning from 01/01/2000 to 12/31/2011. We employed this study design [20] primarily because of transient BZRA use and rarity of pneumonia events, which were not suitable for implementing a cohort study design. The LHID is a subset of the National Health Insurance Research Database (Taiwan National Health Insurance Research Database, 2017) [21], which contains patients’ demographics, medical diagnoses, procedures, and prescription drugs from one million randomly-selected beneficiaries in the universal and compulsory national health insurance, covering more than 99% of 23 million Taiwanese inhabitants. The claims data are quarterly audited by the government [22], and there is high concordance between LHID claims records and patients self-reports [23]. The LHID is frequently analyzed for studying drug safety, including drug-induced pneumonia [24], and patients’ confidentiality is ensured with double-encrypted identifiers. The study protocol was exempt from full review by the institutional review board of Tri-service General Hospital, National Defense Medical Center (B-104-02).

### Selection of study cohort

All newly diagnosed CKD patients aged ≥20 years between 01/01/2001 and 12/31/2010 were identified from the LHID based on *International Classification of Diseases*, *9th Revision*, *Clinical Modification* (ICD-9-CM) codes: 250.4x, 274.1x, 283.11, 403.1x, 404.2x, 404.3x, 440.1x, 442.1x, 447.3x, 572.3x, 580.xx-588.xx, 642.1x, and 646.2x [25] from one inpatient visit or two outpatient or emergency room (ER) visits within 90 days. The adopted codes for identifying CKD patients exhibited high sensitivity and specificity [25,26]. Additionally, these patients must have no kidney-related diagnosis nor undergo renal dialysis or transplantation before the cohort entry date, referred as the second CKD-related outpatient/ER visit or the discharge date from the CKD hospitalization. We further excluded CKD patients without a 1-year continuous National Health Insurance (NHI) enrollment history or those with any pneumonia diagnosis in the year before cohort entry. The final CKD cohort was followed until diagnosis of pneumonia, receipt of renal dialysis or transplantation, disenrollment of the NHI program, death or the end of the study (12/31/2011), whichever occurred first. Death was determined with discharge status from hospitalization claims and eligibility records indicating permanent withdrawal from the NHI. The disenrollment notice must be declared within three days after death by the Taiwan laws (National health Insurance Administration, Ministry of Health and Welfare, 2013) [27]. The operational definitions of selection criteria were detailed in S1 Table.

### Case ascertainment and control selection

During the follow-up of the CKD cohort, we identified cases of pneumonia [28] as patients having two consecutive outpatient visits within 14 days or from one inpatient visit or an ER visit with any diagnosis of pneumonia (ICD-9 codes 480.xx-486.xx, 507.xx), accompanied with chest x-ray procedure codes and prescriptions of respiratory antibiotics (S1 Table). For patients with multiple episodes of pneumonia, we only analyzed the first pneumonia event. The validated coding algorithms (ICD-9 codes 480.xx-486.xx) [29] and the ICD-9 code 507.xx [24] were employed to capture community-acquired and aspiration pneumonia, respectively. The date of the first pneumonia diagnosis marked the index date.

Up to four controls were randomly selected for each case from the CKD risk sets, matching on sex, age (± 5 years old), cohort entry date (± 180 days), prevalent BZRA use at baseline and presence of CKD-related hospitalization in the year preceding the index date. Cases and controls were excluded if they had lung cancer, human immunodeficiency virus infection, tuberculosis and/or cystic fibrosis, or any solid organ transplantations in the year prior to the index date or no matching pairs (detailed in S1 Table). The same index date of the corresponding cases was assigned to controls.

### Assessment of benzodiazepine and Z-drug use

Based on the dispensing date of the most proximal prescription filling records in the year preceding the index date, use of BZDs and Z-drugs was measured and separately classified into current (within 1–30 days), recent (31–90 days), past (91–180 days), and remote use (181–365 days) for case and control groups. Current users were further classified as new users if they did not have any prescription records of BZRAs in the 31 to 365 days preceding the index date, otherwise the remaining current users were categorized as prevalent users. For all analyses, the reference group included CKD patients without any BZRA use in the year preceding the index date.

Different usages of BZRAs were further measured among current users. The mean daily dose of BZDs and Z-drugs were calculated in terms of defined daily dose (DDD) (WHO Collaborating Centre for Drug Statistics Methodology, 2017) [30], and categorized into ≤ 0.5 DDD (low dose), 0.51–1.00 DDD (medium dose), and > 1.00 DDD (high dose). Duration of therapy was categorized as receiving 1–30, 31–90, 91–180, and 181–365 days of therapy, respectively, with permission of a 14-day grace period between successive prescriptions. Oral, injection, or both routes were considered, as well as individual BZRA agents with sufficient samples.

Use of antipsychotics was additionally measured to serve as a positive control given its known risk for pneumonia [31]. Use of buspirone (an anxiolytics) [32] and doxepine (an alternative treatment for insomnia) [33], was measured as a negative control.

### Measurement of covariates

We considered confounders that associate with BZRA use and/or pneumonia risk in both cases and controls. Specifically, we measured demographic characteristics, healthcare use, comorbid conditions and prescribed comedications during the 12 months before the index date, except that respiratory antibiotics were measured in the 15 to 365 days before the index date to avoid capturing the use for early treatment of pneumonia. Covariates were also detailed in the S1 Table.

### Statistical analysis

The incidence rate of pneumonia was estimated based on the Poisson distribution, with 95% confidence intervals (CIs). Conditional logistic regression analyses were performed to estimate odds ratios (ORs) of pneumonia from use of BZRAs with and without adjustments for covariates with *p* < 0.05 in a univariate analysis. Two-sided *p* <0.05 was considered statistically significant. Sample size calculations (S1 Method) indicated that we required 223 cases with 892 controls and 1,221 cases with 4,884 controls for examining current BZD and Z-drug use, respectively. Data cleaning and statistical analyses were conducted using SAS version 9.2 (SAS Institute, Cary, NC, USA), and STATA version 11 (STATA, College, Station, TX, USA), respectively. For data cleaning, we employed range checking and checked for duplicate records and missing data for the analyzed claims records. Only 512 out of 169,249,507 records from the file of ambulatory care expenditures by visits were duplicated, and were excluded accordingly. We retrieved the information on age and sex from outpatient claims, and the demographic data were cross-checked with those from the file of registry for beneficiaries.

We calculated numbers needed to harm (NNTH) [34] for each significant OR >1 based on the following formula: NNTH = 1/[(OR-1) × UER], where OR and UER represent odds ratio and the unexposed event rate of pneumonia within a 30-day period (0.0067 events per 30 days from our analysis).

### Additional analysis

Subgroup analyses included care type of pneumonia (inpatient, outpatient, and ER visit), presence of diabetes mellitus or COPD, and incident and prevalent BZRA users. Multiple sensitivity analyses were additionally conducted. First, we adopted a case-only case-crossover study, in which BZRA use during a time period immediately before the pneumonia event was compared with use in an earlier period to avoid time-invariant confounding and selection bias (S2 Method and S1 Fig). Second, we redefined outcomes with a primary diagnosis of pneumonia. Third, we excluded patients with severe CKD and those initiating BZRAs within 3 days before the pneumonia event to minimize selection bias and protopathic bias, respectively. Severe CKD patients were defined as those dispensed erythropoiesis stimulating agents, which were reimbursed by the NHI only for patients with serum creatinine levels higher than 6 mg/dL [25]. Fourth, cases with aspiration pneumonia were excluded. Fifth, patients hospitalized in the 14 days before the index date were also excluded for avoiding hospital-acquired pneumonia. Sixth, we sub-categorized current users into groups with BZRA prescriptions filled between 1–7, 8–15, or 16–30 days before the index date. Seventh, we adjusted for all covariates listed in Table 1.

**Table 1:** Clinical characteristics between cases and matched controls.

Characteristics			Cases (N = 4,533)	Controls (N = 16,388)	Crude OR (95%CI)	*P*-value^a^
Age,year (Mean±SD)^b^			70.2±13.2	69.5±12.3	NA	NA
Sex, Male, n (%)^b^			2,697 (59.5)	9,721 (59.3)	NA	NA
Hospitalization of CKD in the year before the index date^b^			2,420 (53.4)	8,430 (51.4)	NA	NA
Prevalent use of different types of BZRAs in the year before the cohort entry date^b^		BZD	2,211 (48.8)	8,248 (50.3)	NA	NA
		Z-drug	78 (1.7)	139 (0.8)	NA	NA
		BZD plus Z-drug	675 (14.9)	2,195 (13.4)	NA	NA
Demographic characteristics	Socio-economic status,(NT$/month), n (%)	<20,000	3,253 (71.8)	11,979 (73.1)	Reference	Reference
		20000–39,999	1,080 (23.8)	3,389 (20.7)	1.20 (1.10–1.30)	<0.001
		≥40,000	200 (4.4)	1,020 (6.2)	0.75 (0.63–0.88)	<0.001
	Geographic region	Northern	1,826 (40.3)	6,763 (41.3)	Reference	Reference
		Middle	801 (17.7)	3,142 (19.2)	0.93 (0.85–1.02)	0.14
		Southern	1,576 (34.8)	5,561 (33.9)	1.06 (0.98–1.14)	0.16
		Eastern or other islands	330 (7.3)	922 (5.6)	1.33 (1.16–1.53)	<0.001
Healthcare use^c^	Hospital level	Clinic	1,712 (37.8)	7,314 (44.6)	Reference	Reference
		District hospital	888 (19.6)	2,576 (15.7)	1.44 (1.31–1.59)	<0.001
		Regional hospital	1,055 (23.3)	3,487 (21.3)	1.29 (1.18–1.41)	<0.001
		Medical center	878 (19.4)	3,011 (18.4)	1.25 (1.13–1.37)	<0.001
	No. of outpatient visits	≤19 (Lowest)	1,281 (28.3)	4,565 (27.9)	Reference	Reference
		20–32	975 (21.5)	3,974 (24.2)	0.87 (0.79–0.96)	0.005
		33–50	1,062 (23.4)	3,957 (24.1)	0.94 (0.85–1.04)	0.27
		>51 (Highest)	1,215 (26.8)	3,892 (23.7)	1.09 (0.98–1.21)	0.096
	No. of CKD diagnoses^d^	None	2,307 (50.9)	9,168 (55.9)	Reference	Reference
		1–6	1,096 (24.2)	3,384 (20.7)	1.37 (1.25–1.50)	<0.001
		≥7	1,130 (24.9)	3,836 (23.4)	1.23 (1.13–1.33)	<0.001
Any hospitalization within 14 days before the index date			544 (12.0)	584 (3.6)	3.80 (3.34–4.33)	<0.001
Comorbidities, n (%)^c^	Cardiovascular disease	Hypertension	2,988 (65.9)	10,833 (66.1)	0.97 (0.91–1.05)	0.49
		Diabetes mellitus	2,207 (48.7)	6,958 (42.5)	1.31 (1.23–1.41)	<0.001
		Cerebrovascular disease	1,460 (32.2)	3,370 (20.6)	1.85 (1.72–2.00)	<0.001
		Ischemic heart disease	1,304 (28.8)	4,559 (27.8)	1.02 (0.95–1.11)	0.54
		Heart failure	751 (16.6)	1,747 (10.7)	1.64 (1.49–1.80)	<0.001
		Coronary revascularization	88 (1.9)	330 (2.0)	0.92 (0.72–1.17)	0.48
	Lung disease	COPD	1,018 (22.5)	2,181 (13.3)	1.92 (1.76–2.09)	<0.001
		Asthma	494 (10.9)	1,008 (6.2)	1.86 (1.66–2.09)	<0.001
	Gastrointestinal disease	Gastroesophageal reflux disease	387 (8.5)	1,274 (7.8)	1.08 (0.96–1.22)	0.19
		Swallowing dysfunction	55 (1.2)	78 (0.5)	2.39 (1.68–3.40)	<0.001
	Neurologic disorders	Dementia	548 (12.1)	929 (5.7)	2.22 (1.98–2.50)	<0.001
		Parkinson disease	254 (5.6)	643 (3.9)	1.40 (1.20–1.63)	<0.001
		Epilepsy	154 (3.4)	259 (1.6)	2.11 (1.72–2.58)	<0.001
	Psychiatric disease	Depression	276 (6.1)	888 (5.4)	1.09 (0.94–1.25)	0.25
		Bipolar disorder	47 (1.0)	119 (0.7)	1.30 (0.91–1.84)	0.14
		Schizophrenia	40 (0.9)	86 (0.5)	1.59 (1.08–2.32)	0.018
	No. of anxiety diagnoses	0 (Lowest)	4,043 (89.2)	14,374 (87.7)	Reference	Reference
		1–3	270 (6.0)	1,061 (6.5)	0.88 (0.76–1.01)	0.18
		4–6	84 (1.9)	372 (2.3)	0.76 (0.59–0.96)	0.022
		>6 (Highest)	136 (3.0)	581 (3.5)	0.81 (0.67–0.98)	0.034
	No. of insomnia diagnoses	0 (Lowest)	3,662 (80.8)	13,037 (79.6)	Reference	Reference
		1–3	451 (9.9)	1,674 (10.2)	0.93 (0.83–1.04)	0.063
		4–6	145 (3.2)	608 (3.7)	0.80 (0.67–0.97)	0.024
		>6 (Highest)	275 (6.1)	1,069 (6.5)	0.86 (0.74–0.99)	0.034
	Chronic liver disease		559 (12.3)	1,950 (11.9)	1.06 (0.95–1.17)	0.29
	Cancer (except for lung cancer)		555 (12.2)	1,656 (10.1)	1.22 (1.10–1.35)	<0.001
Comedication, n (%)^c^	Cardiovascular drugs	Diuretics	2,713 (59.8)	7,932 (48.4)	1.64 (1.53–1.76)	<0.001
		CCBs	2,572 (56.7)	8,848 (54.0)	1.11 (1.04–1.19)	0.003
		*β*-blockers	1,851 (40.8)	6,467 (39.5)	1.04 (0.97–1.12)	0.23
		ARBs	1,589 (35.1)	5,724 (34.9)	1.00 (0.93–1.08)	0.97
		ACEIs	1,266 (27.9)	4,145 (25.3)	1.14 (1.05–1.22)	0.001
		Statins	952 (21.0)	3,744 (22.8)	0.90 (0.83–0.98)	0.015
	Gastric acid suppressants	H_2_-blockers	1,524 (33.6)	4,997 (30.5)	1.15 (1.07–1.24)	<0.001
		PPIs	913 (20.1)	2,447 (14.9)	1.43 (1.31–1.56)	<0.001
	Corticosteroids	Systematic	1,804 (39.8)	5,377 (32.8)	1.36 (1.26–1.46)	<0.001
		Inhaled	216 (4.8)	381 (2.3)	2.11 (1.78–2.51)	<0.001
		Topical	2,654 (58.5)	9,455 (57.7)	1.02 (0.95–1.09)	0.66
	Anti-inflammatory drugs	COX-2 selective NSAIDs	538 (11.9)	2,251 (13.7)	0.82 (0.74–0.91)	<0.001
		Nonselective NSAIDs	3,301 (72.8)	12,515 (76.4)	0.81 (0.75–0.87)	<0.001
		Aspirin	2,055 (45.3)	6,489 (39.6)	1.26 (1.17–1.35)	<0.001
	Psychotropic drugs	Antipsychotics	1,213 (26.8)	2,949 (18.0)	1.67 (1.54–1.81)	<0.001
		Antidepressants	915 (20.2)	2,875 (17.5)	1.16 (1.06–1.26)	0.001
		Antiepileptics	939 (20.7)	2,670 (16.3)	1.33 (1.22–1.44)	<0.001
		Anxiolytics^e^	673 (14.8)	2,638 (16.1)	0.89 (0.81–0.98)	0.016
		Sedatives^e^	1,128 (24.9)	3,549 (21.7)	1.19 (1.09–1.28)	<0.001
	Respiratory antibiotics agents		2,974 (65.6)	9,641 (58.8)	1.49 (1.37–1.62)	<0.001
	Opioids		1,917 (42.3)	5,864 (35.8)	1.33 (1.23–1.42)	<0.001
	Influenza & Pneumonia vaccines^f^		1,090 (24.0)	4,532 (27.7)	0.80 (0.73–0.86)	<0.001
	Lung injuring drugs		297 (6.6)	656 (4.0)	1.66 (1.44–1.92)	<0.001
	ESA		114 (2.5)	122 (0.7)	3.41 (2.63–4.42)	<0.001
	Immunosuppressants		53 (1.2)	123 (0.8)	1.62 (1.17–2.25)	0.004

OR, odds ratio; CI, confidence interval; SD, standard deviations; CKD, chronic kidney disease; BZRAs, benzodiazepine receptor agonists; BZDs, benzodiazepines; COPD, chronic obstructive pulmonary disease, CCBs, calcium channel blockers; ARBs, angiotensin II receptor blockers; ACEIs, angiotensin-converting enzyme inhibitors; PPIs, proton pump inhibitors; COX-2, cyclooxygenase-2; NSAIDs, non-steroidal anti-inflammatory drugs; ESAs, erythropoietin stimulating agents.

^a^*P*-value was obtained by conditional logistic regression.

^b^Matching variables.

^c^Measured in the year preceding index date.

^d^The mean number of CKD diagnosis was 6.

^e^Anxiolytics and sedatives did not include benzodiazepine receptor agonists.

^f^Measured in the 15 to 365 days before the index date.

## Results

The study cohort comprised 36,880 newly-diagnosed CKD patients without renal dialysis/renal transplantation, with a mean age of 59.6 years (standard deviation [SD] 16.3), and 55.5% were men (S2 Fig). During the mean 4.9 years (SD 3.2) of follow-up, we identified 5,129 pneumonia cases, corresponding to an incidence rate of 29.6 per 1,000 (95% confidence interval [CI] 28.8–30.4) person-years. After applying the exclusion criteria, a total of 4,533 cases and 16,388 matched controls were analyzed.

Cases and controls were comparable in the matching variables, presence of hypertension, ischemic heart disease, depression and receipt of angiotensin receptor blockers and *β*-blockers (Table 1). Patients with pneumonia, however, were more likely than controls to have majority of the examined comorbidities, and receive medical care and comedications. Conversely, controls were more prevalent to have > 4 visits for insomnia or anxiety in the year, receive statins, anxiolytics (excluding BZRAs), nonsteroidal anti-inflammatory drugs and influenza/pneumonia vaccines. Most of the differences between the two groups were less than 5%. Additionally, the clinical characteristics between the case and control time periods in the case-crossover design are shown in S2 Table.

Table 2 indicates that current use of BZDs was associated with a significantly increased risk of pneumonia (adjusted OR 1.31, 95% CI 1.18–1.46) compared with nonuse, in which a 2.47-fold (95% CI, 2.02–3.03) increased risk was noted with new initiation. Nonetheless, the increased risk was absent with recent, past, remote use of BZDs. On the other hand, new use of Z-drugs and new receipt of combined BZD/Z-drug therapy yielded a 2.94-fold (95% CI 1.65–5.26) and 2.47-fold (95% CI 1.10–5.51) increased risk, respectively. As expected, current use of positive control agents (adjusted OR 1.96, 95% CI 1.54–2.49), but not negative control agents (adjusted OR 0.81, 95% CI 0.35–1.88), was associated with an increased pneumonia risk.

**Table 2:** Risk of pneumonia associated with different types of BZRAs, stratified by recency^a^.

	Cases (N = 4,533)	Controls (N = 16,388)	Crude OR (95%CI)	Adjusted OR^b^ (95%CI)
**Nonuse of BZRA, n (%)**	1,766 (39.0)	6,862 (41.9)	Reference	Reference
**BZD, n (%)**				
Current	973 (21.5)	2,717 (16.6)	1.42 (1.29–1.57)^c^	1.31 (1.18–1.46)^c^
New use	214 (4.7)	298 (1.8)	2.85 (2.36–3.43)^c^	2.47 (2.02–3.03)^c^
Prevalent use	759 (16.7)	2,419 (14.8)	1.21 (1.09–1.34)^c^	1.09 (0.97–1.22)
Recent	371 (8.2)	1,255 (7.7)	1.16 (1.02–1.32)^c^	1.03 (0.89–1.19)
Past	232 (5.1)	1,090 (6.7)	0.82 (0.71–0.96)^c^	0.76 (0.64–0.90)^c^
Remote	269 (5.9)	1,382 (8.4)	0.76 (0.65–0.87)^c^	0.73 (0.62–0.85)^c^
**Z-drug, n (%)**				
Current	77 (1.7)	254 (1.6)	1.11 (0.85–1.46)	1.07 (0.80–1.44)
New use	27 (0.6)	31 (0.2)	3.18 (1.87–5.39)^c^	2.94 (1.65–5.26)^c^
Prevalent use	50 (1.1)	223 (1.4)	0.80 (0.58–1.10)	0.76 (0.54–1.07)
Recent	25 (0.6)	93 (0.6)	1.05 (0.67–1.64)	1.07 (0.66–1.74)
Past	15 (0.3)	77 (0.5)	0.71 (0.41–1.25)	0.78 (0.43–1.41)
Remote	20 (0.4)	139 (0.9)	0.52 (0.32–0.83)^c^	0.49 (0.30–0.81)^c^
**BZD plus Z-drug or switch use, n (%)**				
BZD plus Z-drug				
Current	234 (5.2)	669 (4.1)	1.33 (1.13–1.58)^c^	1.15 (0.94–1.41)
New use	13 (0.3)	16 (0.1)	3.56 (1.68–7.54)^c^	2.47 (1.10–5.51)^c^
Prevalent use	221 (4.9)	653 (4.0)	1.22 (1.03–1.46)^c^	0.99 (0.81–1.22)
Recent	181 (4.0)	480 (2.9)	1.45 (1.20–1.75)^c^	1.20 (0.96–1.50)
Past	116 (2.6)	476 (2.9)	0.93 (0.75–1.16)	0.86 (0.67–1.10)
Remote	182 (4.0)	690 (4.2)	1.00 (0.83–1.20)	0.97 (0.79–1.20)
Switch between BZRA	72 (1.6)	204 (1.2)	1.36 (1.03–1.80)^c^	1.24 (0.91–1.69)

OR, odds ratio; CI, confidence interval; BZRAs, benzodiazepine receptor agonists; BZD, benzodiazepine.

^a^Recency of BZRAs treatment was classified based on the start of the supply of the most recent BARAs prescription before the index date. New users were defined as patients with a prescription that started within 30 days, but did not have any record of BARAs in the 31 to 365 days preceding the index date; the remaining current users were defined as prevalent users.

^b^Adjusted for all confounders with P-value <0.05 listed in Table 1.

^c^P-value <0.05.

Risks of pneumonia related to dose, duration and route of current BZRA therapy were revealed in Table 3. There was a 25%, 28% and 38% significant increase in the risk of pneumonia with BZDs currently being prescribed at a daily dose of ≤ 0.5 DDD, 0.51–1 DDD and > 1 DDD, respectively, indicating a dose-independent association. However, a 1.65-fold (95% CI 1.44–1.89) pneumonia risk incurred with less than 30-day BZD use, but disappeared with prolonged use. The adjusted ORs for parenteral and oral administration of BZDs were 2.88 (95% CI 1.87–4.42) and 1.23 (95% CI 1.10–1.37), respectively. Regarding Z-drug usages, receipt of therapy for 1 to 30 days was the only condition that showed an elevated pneumonia risk (adjusted OR 1.75, 95% CI, 1.13–2.72).

**Table 3:** Risk of pneumonia with current use of different types of BZRAs, stratified by the characteristics and administrations.

	Cases (N = 4,533)	Controls (N = 16,388)	Crude OR (95%CI)	Adjusted OR^a^ (95%CI)
**Non user, n (%)**	1,766 (39.0)	6,862 (41.9)	Reference	Reference
**Current BZD use, n (%)**				
**By dose**				
≦0.50 DDD	490 (10.8)	1,396 (8.5)	1.39 (1.23–1.56)^b^	1.25 (1.09–1.43)^b^
0.51–1.00 DDD	298 (6.6)	821 (5.0)	1.43 (1.24–1.66)^b^	1.28 (1.09–1.50)^b^
>1.00 DDD	185 (4.1)	500 (3.1)	1.44 (1.21–1.73)^b^	1.38 (1.13–1.69)^b^
**By duration**				
1–30 days	520 (11.5)	1,114 (6.8)	1.83 (1.63–2.07)^b^	1.65 (1.44–1.89)^b^
31–90 days	147 (3.2)	489 (3.0)	1.17 (0.96–1.42)	0.98 (0.79–1.22)
91–180 days	88 (2.0)	333 (2.0)	1.04 (0.81–1.33)	0.96 (0.74–1.25)
181–365 days	218 (4.8)	781 (4.8)	1.08 (0.91–1.27)	1.00 (0.83–1.20)
**By route**				
Oral	877 (19.4)	2,623 (16.0)	1.31 (1.19–1.45)^b^	1.23 (1.10–1.37)^b^
Parenteral	60 (1.3)	47 (0.3)	5.05 (3.42–7.46)^b^	2.88 (1.87–4.42)^b^
Oral & Parenteral	36 (0.8)	47 (0.3)	2.81 (1.80–4.39)^b^	1.62 (0.98–2.69)
**Current Z-drug use, n (%)**^c^				
**By dose**				
≤0.50 DDD	11 (0.2)	26 (0.2)	1.59 (0.78–3.24)	1.49 (0.69–3.23)
0.51–1.00 DDD	45 (1.0)	172 (1.1)	0.93 (0.66–1.31)	0.92 (0.63–1.33)
>1.00 DDD	21 (0.5)	56 (0.3)	1.39 (0.83–2.33)	1.20 (0.69–2.11)
**By duration**				
1–30 days	39 (0.9)	77 (0.5)	1.78 (1.19–2.67)^b^	1.75 (1.13–2.72)^b^
31–90 days	13 (0.3)	51 (0.3)	0.96 (0.52–1.78)	0.89 (0.46–1.71)
91–180 days	6 (0.1)	23 (0.1)	0.94 (0.37–2.35)	0.84 (0.32–2.22)
181–365 days	19 (0.4)	103 (0.6)	0.65 (0.39–1.07)	0.60 (0.35–1.04)
**Current BZD plus Z-drug use, n (%)**				
**By dose**				
≤0.50 DDD	12 (0.3)	35 (0.2)	1.32 (0.68–2.58)	0.97 (0.46–2.04)
0.51–1.00 DDD	61 (1.4)	139 (0.9)	1.72 (1.26–2.35)^b^	1.32 (0.93–1.87)
>1.00 DDD	161 (3.6)	495 (3.0)	1.18 (0.97–1.44)	1.03 (0.82–1.29)
**Current BZD plus Z-drug use, n (%)**				
**By duration**				
1–30 days	51 (1.1)	112 (0.7)	1.73 (1.23–2.43)^b^	1.40 (0.95–2.05)
31–90 days	61 (1.4)	124 (0.8)	1.82 (1.32–2.51)^b^	1.40 (0.97–2.01)
91–180 days	40 (0.9)	122 (0.7)	1.21 (0.84–1.76)	1.09 (0.73–1.65)
181–365 days	82 (1.8)	311 (1.9)	0.93 (0.72–1.21)	0.76 (0.57–1.02)
**By route**				
Oral	198 (4.4)	636 (3.9)	1.16 (0.97–1.39)	1.02 (0.83–1.25)
Oral & Parenteral	36 (0.8)	33 (0.2)	3.86 (2.38–6.26)^b^	1.93 (1.12–3.35)^b^

OR, odds ratio; CI, confidence interval; BZRAs, benzodiazepine receptor agonists; BZD, benzodiazepine.

^a^Adjusted for all confounders with P-value <0.05 listed in Table 1.

^b^P-value < 0.05.

^c^Only oral formulation is available for Z-drug.

**Fig 1:**
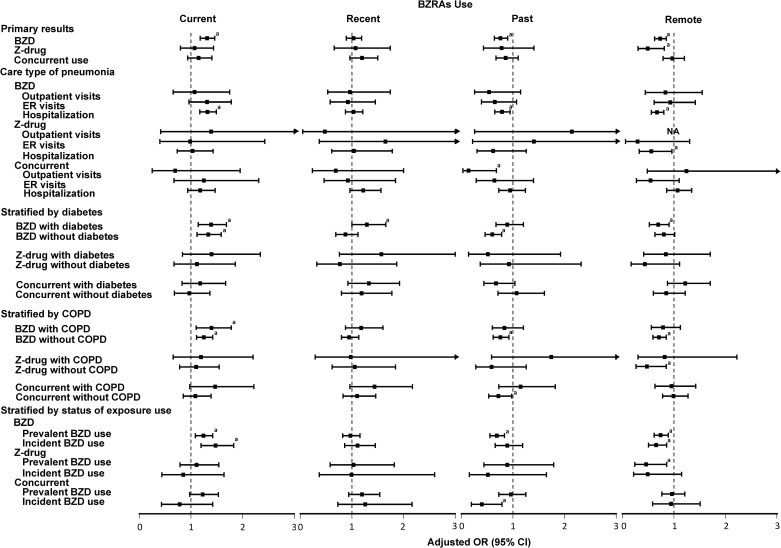
Subgroup analysis for the risk for pneumonia from different BZRA types stratified by recency. BZDs, benzodiazepine; BZRAs, benzodiazepine receptor agonists; NA, not available. ^a^*p*-value <0.05.

Examination of the risk of pneumonia from individual BZRAs indicated that current use of lorazepam, diazepam, chlordiazepoxide, flunitrazepam, midazolam, and nordazepam was associated with a 1.28 to 3.69-fold increased risk of pneumonia (S3 Table). In subgroup analyses (Fig 1 and S4 Table), the risk of pneumonia from current BZD use was confined to hospitalized events, and a higher risk was observed in incident users than prevalent users. Nevertheless, the risk was not varied by presence of COPD or diabetes mellitus, and the risk from current Z-drug use was absent across all subgroups.

**Fig 2:**
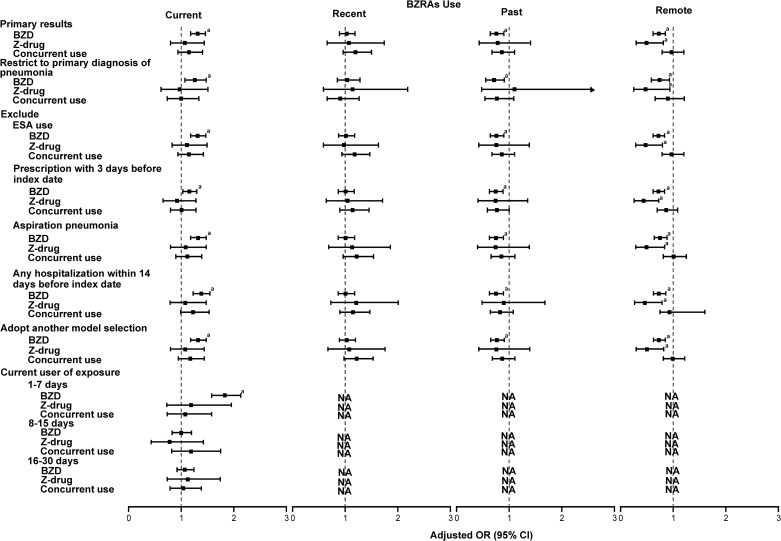
Sensitivity analyses for examining association between risk of pneumonia and type of BZRs under different recency of medication use. NA, not available. ^a^*p*-value <0.05.

Fig 2 indicates that current use of BZDs was associated with a 36% increase in the risk of pneumonia (adjusted OR, 1.36; 95% CI, 1.16–1.60), and no increased risk was found with current non-BZD use (adjusted OR, 1.06; 95% CI, 0.76–1.48) in a case-crossover study, which showed robustness to the main findings. The majority of other sensitivity analyses were consistent with the main findings, such as analyses that examined primary diagnosis of pneumonia only and excluded aspiration pneumonia (Fig 2). S5 Table shows the NNTH for current BZD use in the past 30 days was 478 (95% CI 322–823); for new BZD use was 101 (95% CI 73–145); and for new use of Z-drugs was 76 (95% CI 35–228).

## Discussion

In this large nested case-control study of more than 36,000 CKD patients, risks of pneumonia were found with specific BZRA usages. Current use of BZDs was associated with a significantly increased pneumonia risk by 31%, independent of daily dose, but the risk was doubled or greater when BZDs were newly initiated within 30 days or administered parenterally. New initiation of Z-drugs within 30 days was also found to triple the risk. To our knowledge, this is the first population-based study that examines the risk of pneumonia from various conditions of BZRA use in a CKD population.

As new information, we discovered that the relationship of BZRAs and pneumonia was mainly modified by timing, duration and route of administration. We are the first to report an increased risk of pneumonia associated with new use of BZRAs but not with prevalent use of the medications. This finding may reflect an acute onset of pneumonia from BZRAs and development of tolerance to the risk with longer therapy of the medications. Although confounding cannot be ruled out for the observed phenomenon, confounding is believed not to be differential between new and prevalent users. Additionally, the acute onset of pneumonia from BZRAs is supported by animal studies [7,35], which reported impaired immune cells and increased bacterial count within 1–14 day of BZD use. Furthermore, we observed a higher risk of pneumonia with parenteral BZDs than oral BZDs, which may be attributable to a high injection rate that has been proposed for BZD-induced respiratory adverse events [36]. Alternatively, the propylene glycol contained in parenteral formulations could cause immunosuppression, making patients more susceptible to pneumonia [37]. We also observed that the increased risk of pneumonia persisted across varying levels of BZD daily doses. Accordingly, physicians need to be vigilant with any symptoms of pneumonia when prescribing BZDs at any dose in CKD patients. If CKD patients who receive BZDs and subsequently develop pneumonia, the best approach is to withdraw BZDs rather than to tapper down the dose, which is also supported by the fact that we found recent and past use were not associated with pneumonia risk.

Although some preliminary studies had broadly investigated benzodiazepine adverse events [10,11] or pneumonia risk factors [12,13], their findings could not compare directly with ours. The reasons were because the prior studies did not specifically examine BZRA-associated pneumonia, and they usually had small sample sizes [12,13] and inadequate control for important confounders such as severity for psychotic disorders [11–13] and use of immunosuppressive medications [10,11]. Despite the small samples and survey nature of the study, Dublin et al [15] conducted the first study that directly assessed the relationship between pneumonia and BZRAs, but reported no association. Conversely, Obiora et al [14] found an approximately 2-fold increase in the pneumonia risk with exposure to BZDs or zopiclone within 30 days of therapy. However, their studies were limited by uncertainty of pneumonia diagnosis, mixed effects of BZD and Z-drug use, and selection bias from examinations of a diverse population. Additionally, protopathic bias could also account for their findings [14], in which pneumonia prodromes such as insomnia or anxiety may promote the prescriptions of BZRAs.

The current study attempted to address the limitations of the previous studies. Unlike previous studies [10,11,14] that might have imprecisely defined pneumonia solely by disease coding [38], our pneumonia cases were identified with a set of validated diagnosis codes [29] plus use of antibiotic treatments and chest x-ray procedures. Additionally, the present study had sufficient statistical power, and minimal misclassification of exposure given BZRA prescription filling records were examined from all medical care settings. Moreover, we measured incident events of pneumonia to ensure that BZRA use preceded pneumonia occurrence. We also addressed protopathic bias by disregarding any records of BZRAs in the three days before the index date that could be prescribed for acute early symptoms of pneumonia, but the results remained robust. Finally, we considered numerous important immunomodulatory confounders, and selected cases and controls from a CKD population to minimize selection bias.

Though exact mechanisms are unclear, BZRAs are thought to suppress peripheral immunity through activation of gamma-amino-butyric-acid type A (GABA_A_) receptors [7] or peripheral BZD receptors (PBRs) [39]. Mounting evidence [7,40] has indicated that activation of GABA_A_ receptors may weaken the immune system. Sanders *et al* [7] observed an increased pneumonia mortality rate from diazepam through acting on the α_1_ subunit of GABA_A_ receptors in mice. Similarly, activation of the PBR by BZDs can impair the functions of macrophages [41,42] and neutrophils [41,43–45], though the PBR-signaling pathway has not been verified for pneumonia occurrence. Both mechanisms could underlie the BZD-associated pneumonia because BZDs have high affinities for both types of receptors, whereas the GABAnergic mechanism is probably responsible for Z-drug-induced pneumonia due to their low affinity for PBRs.

When contrasting the pharmacokinetic profiles of individual BZRAs (S6 Table), we found that BZRAs with long half-lives and/or active metabolites may play a pivotal role in the association between pneumonia and BZRA use. Given our CKD population suffered impaired renal functions, and most BZDs are renally excreted, BZDs with active metabolites or long half-lives could accumulate in the body, and subsequently result in pneumonia from unexpectedly high plasma concentrations of BZDs.

Several limitations in the current study need to be addressed. First, confounding by indication bias could threaten our findings, hence we adjusted the main indications of BZRAs in multivariate analyses, and confirmed that the use of a similarly-indicated negative control agent had no association with pneumonia. However, residual confounding could exist given that indications of BZD/Z-drug use may not have been completely recorded in the analyzed database due to limited three and five disease codes that could be made in each outpatient and inpatient visit, respectively. This insufficient data record probably affected our results minimally because present evidence has not linked the main indications of BZD/Z-drug use, including insomnia and anxiety, with pneumonia. Second, despite our extensive matching scheme and confounder adjustment, our findings may still be confounded by CKD severity and residual confounding. However, the analyzed subjects were believed to come from a relatively homogeneous stage 4 CKD population, given we had excluded predialysis and dialysis patients, and used a validated coding scheme [25,26] that detected patients with an eGFR ≤ 45 mL/min/1.74m. Additionally, the main findings persisted in the alternative case-crossover study, which controlled for time-invarying confounders. Third, inclusion of prevalent BZRA users could potentially underestimate the overall risks since they might have developed tolerance for pneumonia. Fourth, another limitation lies in the unavailability of laboratory data for renal function and chest x-ray results as well as etiology of pneumonia. Fifth, we aimed to assess a high risk population, which could limit the generalizability of our findings.

In conclusion, current use of BZRAs, particularly BZDs, is associated with an increased risk of pneumonia in CKD patients. The risk is especially high when BZDs and/or Z-drugs are newly initiated within 30 days or when BZDs are parentally administered.

## Supporting information

S1 Method Sample size calculation. (DOCX)

S2 Method Case-crossover design. (DOCX)

S1 Table Operational definition of case identification and confounding factors. (DOCX)

S2 Table Clinical characteristic between a case period and control period when adopting a case-crossover design. (DOCX)

S3 Table Risk of pneumonia associated with current use of different types of BZRAs, stratified by individual drug use. (DOCX)

S4 Table Subgroup analysis for the risk of pneumonia in relation to different types of BZRAs, stratified by recency. (DOCX)

S5 Table Numbers needed to harm for BZD- and Z-drug associated pneumonia risk in CKD patients. (DOCX)

S6 Table Pharmacokinetic parameters and characteristics of analyzed individual benzodiazepine receptor agonists (BZRAs). (DOCX)

S1 Fig Graphical presentation of the case-crossover design depicting case, control and washout periods. (DOCX)

S2 Fig Flow diagram depicting selection process of study subjects. (DOCX)
