# Supplementary material for: Benzodiazepine and Z-drug use and risk of pneumonia in patients with chronic kidney disease: A population-based nested case-control study
Source: PLoS One. 2017 Jul 10;12(7):e0179472. doi: 10.1371/journal.pone.0179472 (PMC5503235; doi:10.1371/journal.pone.0179472)
Supplement: S1 Method — (DOCX) [file pone.0179472.s001.docx]

**S1 Method. Sample size calculation**

We used Power and Sample Size software for sample size calculation based on the following conditions: 1) The proportions of CKD patients without pneumonia who currently received BZD and Z-drug were 19% and 2.7%, respectively, calculated during the 30 days after the cohort entry date; 2) an OR of 1.89 was expected for both types of BZRAs;^1^ 3) power was set as 80%; 4) type 1 error of 5%; 5) correlation coefficient of exposure between matched cases and controls was set to be 0.5; and 6) a ratio of 4 controls per each case. A total of 223 cases and 892 controls were required for investigating the risk of pneumonia associated with current BZD use, whereas 1,221 cases and 4,884 controls were needed for current use of Z-drugs.

**Reference:**

1. Obiora E, Hubbard R, Sanders RD, et al. The impact of benzodiazepines on occurrence of pneumonia and mortality from pneumonia: a nested case-control and survival analysis in a population-based cohort. *Thorax.* 2013;68(2):163-170.
