# Supplementary material for: Benzodiazepine and Z-drug use and risk of pneumonia in patients with chronic kidney disease: A population-based nested case-control study
Source: PLoS One. 2017 Jul 10;12(7):e0179472. doi: 10.1371/journal.pone.0179472 (PMC5503235; doi:10.1371/journal.pone.0179472)
Supplement: S2 Method — (DOCX) [file pone.0179472.s002.docx]

**S2 Method. Case-crossover design**

We used a case-crossover design to evaluate the risk of pneumonia associated with current BZRAs in order to eliminate interpersonal variations and time-invariant confounders, such as body mass index, smoking and lifestyle. First, we retrieved all pneumonia cases identified in our nested case control study (n=4,533), and excluded cases whose follow-up period was less than 120 days (n=497). A total of 4,036 cases were eligible in the case-crossover analysis. The index date was referred to the date of incident pneumonia diagnosis. In each case, a case and control period were set on 1 to 30 days and 91-120 days before the index date, respectively, with employment of a 60-day buffer period between the two time periods (depicted in Supplemental Figure 1). Use of BZRAs and important confounders (Supplemental Table 2) were measured in both case and control periods. Conditional logistic regressions were employed to estimate the odds ratios (ORs) of pneumonia risk from current BZRA use with adjustments of significant time-varying confounders (p<0.05) in S2 Table.
