# Supplementary material for: Benzodiazepine and Z-drug use and risk of pneumonia in patients with chronic kidney disease: A population-based nested case-control study
Source: PLoS One. 2017 Jul 10;12(7):e0179472. doi: 10.1371/journal.pone.0179472 (PMC5503235; doi:10.1371/journal.pone.0179472)
Supplement: S1 Table — (DOCX) [file pone.0179472.s003.docx]

**S1 Table. Operational definition of case identification and confounding factors**

| **Items** | **Operational definition** |
| --- | --- |
| **Inclusion criteria** |  |
| Chronic kidney disease | ICD-9 code diagnosis code: 250.4x, 274.1x, 283.11, 403.1x, 404.2x, 404.3x, 440.1x, 442.1x, 447.3x, 572.3x, 580.xx-588.xx, 642.1x, 646.2x |
| Dialysis | ICD-9 code diagnosis code: V45.1x, V56.xx, 585.6x, 996.56, 996.68, 996.73  ICD-9 procedure code: 39.95, 54.98  NHI procedure code: 58001C, 58019C, 58020C, 58021C, 58022C, 58023C, 58024C, 58025C, 58027C, 58029C, 58002C, 58011A, 58011B, 58011C, 58017A, 58017B, 58017C, 58026C, 58028C |
| Renal transplantation | ICD-9 code diagnosis code: V42.0, 996.81  ICD-9 procedure code: 55.6x  NHI procedure code: 76020A, 76020B |
| **Exclusion criteria** |  |
| Lung Cancer | ICD-9 code diagnosis code: 162.xx |
| Human immunodeficiency  Virus | ICD-9 code diagnosis code: 042.xx, V08.xx |
| Tuberculosis | ICD-9 code diagnosis code: 010.xx-018.xx |
| Cystic fibrosis | ICD-9 code diagnosis code: 277.0x |
| Solid organ transplantation | ICD-9 code diagnosis code: 996.8x (except for 996.81), V42.1x, V42.6x, V42.7x, V42.8x, V42.9x  ICD-9 procedure code:33.5x, 33.6x, 37.5x, 50.5x, 52.8x |
| **Case Identification** | |
| Pneumonia | ICD-9 code diagnosis code: 480.xx, 481.xx, 482.xx, 483.xx, 484.xx,  485.xx, 486.xx, 507.xx |
| Respiratory antibiotics | Amikacin, amoxicillin, ampicillin, azithromycin, aztreonam, bacampicillin, benzylpenicillin, cefaclor, cefadroxil, cefamandole, cefatrizine, cefazolin, cefepime, cefixime, cefmenoxime, cefmetazole, cefonicid, cefoperazone, cefotaxime, cefoxitin, cefpirome, cefpodoxime, cefradine, ceftazidime, ceftibuten, ceftizoxime, ceftriaxone, cefuroxime, chlortetracycline, ciprofloxacin, clarithromycin, clindamycin, cloxacillin, dicloxacillin, doxycycline, erythromycin, gemifloxacin, gentamicin, Inipenem, isepamicin, latamoxef, levofloxacin, linezolid, meropenem, metampicillin, minocycline, minocycline, moxifloxacin, ofloxacin, oxacillin, oxacillin, oxytetracycline, penicillin, piperacillin, streptomycin, talampicillin, teicoplanin, tetracycline, tetracycline, ticarcillin, tigecycline, tobramycin, trimethoprim/sulfamethoxazole, vancomycin |
| X-ray | ICD-9 procedure code: 873, 8738, 8739, 874, 8743, 8744, 8749  NHI procedure code: 32001C, 32002C |

**S1 Table. Operational definition of case identification and confounding factors (continued)**

| **Items** | **Operational definition** |
| --- | --- |
| **Comorbidities** |  |
| Cerebrovascular disease | ICD-9 code diagnosis code: 430.xx-438.xx |
| Diabetes mellitus | ICD-9 code diagnosis code: 250.xx |
| Hypertension | ICD-9 code diagnosis code: 401.xx, 402.xx, 403.xx, 404.xx, 405.xx |
| Heart failure | ICD-9 code diagnosis code: 428.xx |
| Ischemic heart disease | ICD-9 code diagnosis code: 410.xx, 411.xx, 412.xx, 413.xx, 414.xx |
| Coronary revascularization | ICD-9-CM procedure codes：36.01, 36.02, 36.05, 36.06, 36.09, 36.10,36.11, 36.12, 36.13, 36.14, 36.15, 36.16, 36.17, 36.19 |
| Asthma | ICD-9 code diagnosis code: 493.xx |
| COPD | ICD-9 code diagnosis code: 491.xx, 492.xx, 496.xx |
| Chronic liver disease | ICD-9 code diagnosis code: 571.xx |
| Cancer  (Except for lung cancer) | ICD-9 code diagnosis code: 140.xx-208.xx (except 162.xx) |
| Gastroesophageal reflux disease | ICD-9 code diagnosis code: 530.81, 530.1x, 530.2x, 530.3x |
| Swallowing dysfunction | ICD-9 code diagnosis code: 787.2x |
| Dementia | ICD-9 code diagnosis code: 290.xx |
| Parkinson disease | ICD-9 code diagnosis code: 332.xx |
| Epilepsy | ICD-9 code diagnosis code: 345.xx, 780.3x |
| Bipolar disorder | ICD-9 code diagnosis code: 296.0, 296.1, 296.4, 296.5, 296.6, 296.7, 296.8 |
| Depression | ICD-9 code diagnosis code: 296.2x, 296.3x, 300.4, 311 |
| Schizophrenia | ICD-9 code diagnosis code: 295.xx |
| Insomnia | ICD-9 code diagnosis code: 307.4x, 780.5x, 327.3, 327.4 |
| Anxiety | ICD-9 code diagnosis code: 300.x (except 300.4) |
| Cancer | ICD-9 code diagnosis code: 140.xx-208.xx (except 162.xx) |

**S1 Table. Operational definition of case identification and confounding factors (continued)**

| **Comedication classes** | **Individual drugs** |
| --- | --- |
| Cardiovascular drugs | |
| ACEIs | Benazepril, captopril, cilazapril, enalapril, fosinopril, imidapril, Indapamide, lisinopril, perindopril, quinapril, ramipril, |
| ARBs | Candesartan, eprosartan, irbesartan, losartan, olmesartan, telmisartan, valsartan |
| β-blockers | Acebutolol, alprenolol, atenolol, betaxolol, bisoprolol, bupranolol, carteolol, carvedilol, clopamide, esmolol, labetalol, metaprolol, metipranolol, metoprolol , nadolol, oxprenolol, pindolol, propranolol, sotalol, timolol |
| CCBs | Amlodipine, barnidipine, benidipine, diltiazem, felodipine, isradipine, lacidipine, lercanidipine, nicardipine, nifedipine, nimodipine, nisoldipine, nitrendipine, verapamil |
| Diuretics | Amiloride, bendroflumethiazide, benzylhydrochlorothiazide, bumetanide, canrenoate, eplerenone, ethacrynic acid, furosemide, hydrochlorothiazide, hydroflumethiazide, indapamide, metolazone, spironolactone, triamterene, trichlormethiazide |
| Statins | Atorvastatin, fluvastatin, lovastatin, pitavastatin, pravastatin, rosuvastatin, simvastatin |
| Gastric acid suppressants | |
| H_2_-blockers | Cimetidine, famotidine, nizatidine, ranitidine, roxatidine |
| PPIs | Esomeprazole, lansoprazole, omeprazole, pantoprazole |
| Corticosteroids |  |
| Systematic | Betamethasone, cortisone, dexamethasone, fludrocortisones, fluocortolone, hydrocortisone, methandrostenolone, methylpredisolone, nandrolone, oxymetholone, paramethasone, phenylbutazone, prednisolone, stanozolol, triamcinolone |
| Inhaled | Budesonide, beclomethasone, ciclesonide, fluticasone |
| Topical | Amcinonide, beclomethasone, budesonide, clobetasol, clobetasone, cortisone, desoximetasone, diflucortolone, fluclorolone, flumethasone, fluocinolone, fluocinonide, fluocortolone, fluticasone, fusidic acid, halcinonide, hydrocortisone, mometasone, nandrolone, prednisolone, prednisolone, tixocortol , triamcinolone |
| Anti-inflammatory drugs | |
| Aspirin | Aspirin |
| NSAIDs |  |
| COX-2 selective | Celecoxib, etoricoxib, rofecoxib, niflumic acid, nimesulide |

**S1 Table. Operational definition of case identification and confounding factors (continued)**

| **Comedication classes** | **Individual drugs** |
| --- | --- |
| Nonselective | Aceclofenac, acemetacin, alclofenac, alminoprofen, benzydamine, diclofenac, diclofenac, etodolac, etofenamate, fenbufen, fenoprofen, flufenamic acid, flurbiprofen, ibuprofen, indomethacin, ketoprofen, meclofenamic acid, mefenamic acid, meloxicam, mepirizole, nabumetone, naproxen, nefopam, phenylbutazone, piroxicam, salsalate |
| Immunosuppressants | Adalimumab, azathioprine, cladribine, cyclosporine, etanercept, fludarabine, golimumab, mercaptopurine, methotrexate, muromonab, mycophenolate, rituximab, sirolimus, tacrolimus, tioguanine |
| Psychotropic drugs |  |
| Antipsychotics | Amisulpride, aripiprazole, chlorpheniramine, chlorpromazine, chlorprothixene, cloapine, clopenthixol, clothiapine, clotiapine, clozapine, droperidol, flupertixol, fluphenazine, haloperidol, levomepromazine, loxapine, methotrimeprazine, moperone, olanzapine, paliperidone, penfluridol, perphenazine, pimozide, pipotiazine, prochlorperazine, quetiapine, risperidone, sulpiride, thioridazine, thiothixene, tiotixene, trifluoperazine, ziprasidone, zotepine, zuclopenthixol |
| Antidepressants | Agomelatine, amitriptyline, bupropion, chlordiazepoxide, citalopram, clomipramine, dothiepin, doxepin, duloxetine, escitalopram, fluoxetine, fluvoxamine, imipramine, maprotiline, melitracen, mianserin, milnacipran, mirtazapine, moclobemide, paroxetine, sertraline, trazodone, venlafaxine, viloxazine |
| Antiepileptics | Carbamazepine, clonazepam, gabapentin, lamotrigine, levetitacetam, oxcarbazepine, phenobarbital, phenytoin, pregabalin, primidone, tiagabine, topiramate, valproic acid, vigabatrin, zonisamide |
| Anxiolytics^a^ | Buspirone, mephenoxalone, meprobamate, hydroxyzine |
| Sedatives^a^ | Alimemazine, carisoprodol, chlormezanone, dexmedetomidine, diphenhydramine, phenprobamate |
| Opioids | Alfentanil, apomorphine, buprenorphine, codeine, fentanyl, hydromorphone, meperidine, morphine, noscapine, opium, tramadol |
| Lung injuring drugs | Amiodarone, amphotericin B, flecainide, methylphenidate, nitrofurantoin, penicillamine, sulfasalazine |
| ESA | Darbepoetin α, epoetinα, epoetin β, methoxy polyethylene glycol-epoetinβ, recombinant human erythropoietin |
| ^a^Anxiolytics and sedatives did not include benzodiazepine receptor agonists.  Abbreviations: ACEIs, angiotensin-converting enzyme inhibitors; ARBs, angiotensin II receptor blockers; CCBs, calcium channel blockers; CKD, chronic kidney disease; COPD, chronic obstructive pulmonary disease; COX-2, cyclooxygenase-2; ESAs, erythropoietin stimulating agents; NSAIDs, non-steroidal anti-inflammatory drugs; PPIs, proton pump inhibitors. | |
