# Supplementary material for: Benzodiazepine and Z-drug use and risk of pneumonia in patients with chronic kidney disease: A population-based nested case-control study
Source: PLoS One. 2017 Jul 10;12(7):e0179472. doi: 10.1371/journal.pone.0179472 (PMC5503235; doi:10.1371/journal.pone.0179472)
Supplement: S2 Table — (DOCX) [file pone.0179472.s004.docx]

**S2 Table. Clinical characteristic between a case period and control period when adopting a case-crossover design**

| **Characteristics** | **Case period**  **N=4,036** | **Control period**  **N=4,036** |
| --- | --- | --- |
| Age (y) | 70.0±0.2 | 70.0±0.2 |
| Sex, Male | 2,400 (59.5) | 2,400 (59.5) |
| Healthcare use |  |  |
| No. of outpatient visits^a^ |  |  |
| ≦4 | 1,206 (30.0) | 1,656 (41.0) |
| 5-7 | 1,649 (40.9) | 1,512 (37.5) |
| ≧8 | 1,181 (29.3) | 868 (21.5) |
| No. of CKD diagnoses^a^ |  |  |
| 0 | 2,954 (73.2) | 3,008 (74.5) |
| 1-3 | 949 (23.5) | 913 (22.6) |
| 4 | 62 (1.5) | 61 (1.5) |
| ≧5 | 71 (1.8) | 54 (1.3) |
| Comorbidities |  |  |
| Cardiovascular disease |  |  |
| Hypertension^a^ | 2,071 (51.3) | 1,956 (48.5) |
| Diabetes mellitus^a^ | 1,681 (41.7) | 1,614 (40.0) |
| Cerebrovascular disease^a^ | 888 (22.0) | 803 (19.9) |
| Ischemic heart disease^a^ | 718 (17.8) | 666 (16.5) |
| Heart failure^a^ | 408 (10.1) | 298 (7.4) |
| Coronary revascularization | 9 (0.2) | 10 (0.2) |
| Lung disease |  |  |
| COPD^a^ | 515 (12.8) | 398 (9.9) |
| Asthma^a^ | 256 (6.3) | 160 (4.0) |
| Gastrointestinal disease |  |  |
| Gastroesophageal reflux disease^a^ | 147 (3.6) | 105 (2.6) |
| Swallowing dysfunction | 21 (0.5) | 14 (0.3) |
| Neurologic disorders |  |  |
| Dementia^a^ | 361 (8.9) | 293 (7.3) |
| Parkinson disease | 170 (4.2) | 158 (3.9) |
| Epilepsy^a^ | 70 (1.7) | 52 (1.3) |
| Psychiatric disease |  |  |
| Depression | 125 (3.1) | 112 (2.8) |
| Bipolar disorder | 26 (0.6) | 30 (0.7) |
| Schizophrenia | 27 (0.7) | 25 (0.6) |
| No. of anxiety diagnoses |  |  |
| 0 | 3,832 (95.0) | 3,837 (95.1) |
| 1-2 | 169 (4.2) | 173 (4.3) |
| ≧3 | 35 (0.9) | 26 (0.6) |

**S2 Table. Clinical characteristic between case period and control period when adopting a case-crossover design (continued)**

| **Characteristics** | | **Case periods**  **N=4,036** | | **Control periods**  **N=4,036** | |
| --- | --- | --- | --- | --- | --- |
| No. of insomnia diagnoses | |  | |  | |
| 0 | | 3,661 (90.7) | | 3,678 (91.1) | |
| 1-2 | | 297 (7.4) | | 267 (6.6) | |
| ≧3 | | 78 (1.9) | | 91 (2.7) | |
| Chronic liver disease | | 244 (6.0) | | 223 (5.5) | |
| Cancer (except for lung cancer)^a^ | | 386 (9.6) | | 317 (7.9) | |
| Comedication | |  | |  | |
| Cardiovascular drugs | |  | |  | |
| CCBs^a^ | | 1,593 (39.5) | | 1,535 (38.0) | |
| Diuretics^a^ | | 1,543 (38.2) | | 1,438 (35.6) | |
| *β*-blockers | | 1,022 (25.3) | | 987 (24.5) | |
| ARBs | | 944 (23.4) | | 950 (23.5) | |
| ACEIs | | 561 (13.9) | | 572 (14.2) | |
| Statins | | 536 (13.3) | | 541 (13.4) | |
| Gastric acid suppressants | |  | |  | |
| H_2_-blockers^a^ | | 502 (12.4) | | 351 (8.7) | |
| PPIs^a^ | | 319 (7.9) | | 201 (5.0) | |
| Corticosteroids | |  | |  | |
| Systematic^a^ | | 639 (15.8) | | 443 (11.0) | |
| Inhale | | 104 (2.6) | | 89 (2.2) | |
| Topical | | 838 (20.8) | | 785 (19.4) | |
| Anti-inflammatory drugs | |  | |  | |
| Nonselective NSAIDs^a^ | | 1,612 (39.9) | | 1,268 (31.4) | |
| COX-2 selective NSAIDs | | 216 (5.4) | | 194 (4.8) | |
| Aspirin^a^ | | 1,087 (26.9) | | 1,035 (25.6) | |
| Psychotropic drugs | |  | |  | |
| Antipsychotics^a^ | | 470 (11.6) | | 362 (9.0) | |
| Antidepressants | | 410 (10.2) | | 390 (9.7) | |
| Antiepileptics^a^ | | 424 (10.5) | | 357 (8.8) | |
| Anxiolytics^b^ | | 165 (4.1) | | 168 (4.2) | |
| Sedatives^a,b^ | | 247 (6.1) | | 177 (4.4) | |
| Respiratory antibiotics^c^ | | 327 (8.1) | | 356 (8.8) | |
| Opioids^a^ | | 686 (17.0) | | 384 (9.5) | |
| Influenza & Pneumonia vaccines^a^ | | 88 (2.2) | | 126 (3.1) | |
| Lung injuring drugs^a^ | | 124 (3.1) | | 103 (2.6) | |
| ESA^a^ | | 77 (1.9) | | 51 (1.3) | |
| Immunosuppressants | | 29 (0.7) | | 29 (0.7) | |
|  | |  | |  | |
| ^a^*P*-value <0.05.  ^b^Anxiolytics and sedatives did not include benzodiazepine receptor agonists.  ^c^Respiratory antibiotics were measured in the 30 to 15 days before the index date.  Abbreviations: ARBs, angiotensin II receptor blockers; ACEIs, angiotensin-converting enzyme inhibitors; CCBs, calcium channel blockers; CKD, chronic kidney disease; COPD, chronic obstructive pulmonary disease; COX-2, cyclooxygenase-2; ESAs, erythropoietin stimulating agents; NSAIDs, non-steroidal anti-inflammatory drugs; PPIs, proton pump inhibitors. | | | | | |
