# Supplementary material for: Benzodiazepine and Z-drug use and risk of pneumonia in patients with chronic kidney disease: A population-based nested case-control study
Source: PLoS One. 2017 Jul 10;12(7):e0179472. doi: 10.1371/journal.pone.0179472 (PMC5503235; doi:10.1371/journal.pone.0179472)
Supplement: S3 Table — (DOCX) [file pone.0179472.s005.docx]

**S3 Table. Risk of Pneumonia Associated with Current Use of Different Types of BZRAs, Stratified by Individual Drug Use**^a^

|  | **Cases**  **(N=4,533)** | **Controls (N=16,388)** | **Crude OR**  **(95%CI)** | **Adjusted OR^b^ (95%CI)** |
| --- | --- | --- | --- | --- |
| **Nonuse of BZRAs, n (%)** | 1,766 (39.0) | 6,862 (41.9) | Reference | Reference |
| **Current use** |  |  |  |  |
| **BZD only** |  |  |  |  |
| Alprazolam | 88 (1.9) | 331 (2.0) | 1.07 (0.84-1.37) | 1.09 (0.84-1.42) |
| Bromazepam | 17 (0.4) | 89 (0.5) | 0.76 (0.45-1.28) | 0.83 (0.47-1.46) |
| Chlordiazepoxide | 119 (2.6) | 316 (1.9) | 1.51 (1.21-1.88)^c^ | 1.55 (1.22-1.96)^c^ |
| Clonazepam | 74 (1.6) | 241 (1.5) | 1.23 (0.94-1.61) | 0.99 (0.73-1.34) |
| Diazepam | 68 (1.5) | 163 (1.0) | 1.63 (1.22-2.18)^c^ | 1.55 (1.14-2.12)^c^ |
| Estazolam | 77 (1.7) | 214 (1.3) | 1.40 (1.06-1.83)^c^ | 1.30 (0.97-1.75) |
| Fludiazepam | 61 (1.3) | 196 (1.2) | 1.23 (0.91-1.65) | 1.13 (0.82-1.56) |
| Flunitrazepam | 18 (0.4) | 39 (0.2) | 1.84 (1.05-3.24)^c^ | 2.29 (1.26-4.18)^c^ |
| Lorazepam | 150 (3.3) | 368 (2.2) | 1.58 (1.29-1.93)^c^ | 1.28 (1.03-1.60)^c^ |
| Midazolam | 41 (0.9) | 51 (0.3) | 3.29 (2.16-5.02)^c^ | 2.43 (1.53-3.86)^c^ |
| Nordazepam | 7 (0.2) | 8 (0.1) | 3.62 (1.30-10.06)^c^ | 3.69 (1.16-11.76)^c^ |
| Oxazolam | 29 (0.6) | 122 (0.7) | 0.92 (0.61-1.39) | 1.02 (0.66-1.57) |
| Triazolam | 9 (0.2) | 20 (0.1) | 1.65 (0.74-3.65) | 1.69 (0.72-3.96) |
| **Z-drug only** |  |  |  |  |
| Zolpidem | 59 (1.3) | 213 (1.3) | 1.00 (0.74-1.35) | 0.95 (0.69-1.32) |
| Zopiclone | 17 (0.4) | 38 (0.2) | 1.59 (0.88-2.87) | 1.63 (0.86-3.09) |

^a^Not all individual BZDs and Z-drugs were analyzed due to small sample sizes.

^b^Adjusted for all confounders listed in Table 1.

^c^*P*-value < 0.05.

Abbreviations: BZD, benzodiazepine; BZRAs, benzodiazepine receptor agonists; CI, confidence interval; OR, odds ratio.
