# Supplementary material for: Benzodiazepine and Z-drug use and risk of pneumonia in patients with chronic kidney disease: A population-based nested case-control study
Source: PLoS One. 2017 Jul 10;12(7):e0179472. doi: 10.1371/journal.pone.0179472 (PMC5503235; doi:10.1371/journal.pone.0179472)
Supplement: S4 Table — (DOCX) [file pone.0179472.s006.docx]

**S4 Table. Subgroup analysises for the risk of pneumonia in relation to different types of BZRAs, stratified by recency**

| **Characteristics** | **Current use** | | **Recent use** | | **Past use** | | **Remote use** | |
| --- | --- | --- | --- | --- | --- | --- | --- | --- |
|  | **Adjusted OR**^a^ **(95% CI)** | **Interaction**  ***P*-value** | **Adjusted OR**^a^ **(95% CI)** | **Interaction**  ***P*-value** | **Adjusted OR**^a^ **(95% CI)** | **Interaction**  ***P*-value** | **Adjusted OR**^a^ **(95% CI)** | **Interaction**  ***P*-value** |
| **Stratified by diabetes** |  |  |  |  |  |  |  |  |
| BZD with diabetes | 1.39 (1.14-1.69)^b^ | 0.45 | 1.29 (1.00-1.66)^b^ | 0.18 | 0.89 (0.67-1.20) | 0.080 | 0.69 (0.52-0.90)^b^ | 0.26 |
| BZD without diabetes | 1.34 (1.12-1.59)^b^ |  | 0.88 (0.69-1.12) |  | 0.60 (0.46-0.78)^b^ |  | 0.80 (0.63-1.01) |  |
| Z-drug with diabetes | 1.40 (0.84-2.34) | 0.40 | 1.57 (0.76-3.24) | 0.082 | 0.52 (0.14-1.92) | 0.67 | 0.84 (0.41-1.70) | 0.55 |
| Z-drug without diabetes | 1.12 (0.67-1.86) |  | 0.77 (0.32-1.87) |  | 0.92 (0.37-2.32) |  | 0.43 (0.17-1.11) |  |
| BZD plus Z-drug with diabetes | 1.18 (0.83-1.67) | 0.63 | 1.33 (0.92-1.92) | 0.97 | 0.67 (0.44-1.04) | 0.074 | 1.22 (0.87-1.70) | 0.27 |
| BZD plus Z-drug without diabetes | 0.97 (0.68-1.37) |  | 1.19 (0.80-1.77) |  | 1.07 (0.71-1.61) |  | 0.85 (0.60-1.22) |  |
| **Stratified by COPD** |  |  |  |  |  |  |  |  |
| BZD with COPD | 1.40 (1.10-1.78)^b^ | 0.46 | 1.18 (0.88-1.60) | 0.63 | 0.84 (0.60-1.20) | 0.69 | 0.79 (0.56-1.12) | 0.81 |
| BZD without COPD | 1.25 (1.11-1.42)^b^ |  | 0.95 (0.80-1.13) |  | 0.76 (0.62-0.92)^b^ |  | 0.71 (0.59-0.85)^b^ |  |
| Z-drug with COPD | 1.20 (0.66-2.20) | 0.87 | 0.98 (0.29-3.27) | 0.85 | 1.74 (0.59-5.15) | 0.28 | 0.82 (0.30-2.22) | 0.37 |
| Z-drug without COPD | 1.10 (0.78-1.55) |  | 1.06 (0.62-1.84) |  | 0.59 (0.28-1.26) |  | 0.47 (0.26-0.85)^b^ |  |
| BZD plus Z-drug with COPD | 1.47 (0.98-2.22) | 0.36 | 1.44 (0.96-2.17) | 0.94 | 1.15 (0.73-1.82) | 0.19 | 0.95 (0.63-1.42) | 0.29 |
| BZD plus Z-drug without COPD | 1.09 (0.85-1.39) |  | 1.10 (0.83-1.46) |  | 0.72 (0.53-0.98)^b^ |  | 0.99 (0.78-1.27) |  |
| **Stratified by status of exposure use** | | | | | | | | |
| Prevalent BZD use | 1.24 (1.09-1.42)^b^ | 0.027 | 0.97 (0.82-1.16) | 0.27 | 0.69 (0.56-0.84)^b^ | 0.12 | 0.74 (0.61-0.89)^b^ | 0.44 |
| Incident BZD use | 1.48 (1.20-1.83)^b^ |  | 1.11 (0.86-1.45) |  | 0.89 (0.66-1.19) |  | 0.65 (0.50-0.86)^b^ |  |
| Prevalent Z-drug use | 1.11 (0.79-1.54) | 0.65 | 1.03 (0.58-1.82) | 0.85 | 0.89 (0.44-1.79) | 0.45 | 0.46 (0.24-0.86)^b^ | 0.84 |
| Incident Z-drug use | 0.85 (0.44-1.64) |  | 0.99 (0.37-2.60) |  | 0.52 (0.16-1.65) |  | 0.49 (0.21-1.15) |  |
| Prevalent BZD plus Z-drug use | 1.23 (0.98-1.53) | 0.52 | 1.20 (0.94-1.54) | 0.37 | 0.96 (0.73-1.25) | 0.048 | 0.96 (0.76-1.21) | 0.68 |
| Incident BZD plus Z-drug use | 0.78 (0.43-1.42) |  | 1.26 (0.73-2.16) |  | 0.40 (0.20-0.79)^b^ |  | 0.94 (0.58-1.51) |  |
| ^a^Adjusted for all covariates with p<0.05 in Table 1.  ^b^*P*-value <0.05.  Abbreviations: BZDs, benzodiazepines; BZRAs, benzodiazepine receptor agonists; COPD, chronic obstructive pulmonary disease. | | | | | | | | |
