# Supplementary material for: Benzodiazepine and Z-drug use and risk of pneumonia in patients with chronic kidney disease: A population-based nested case-control study
Source: PLoS One. 2017 Jul 10;12(7):e0179472. doi: 10.1371/journal.pone.0179472 (PMC5503235; doi:10.1371/journal.pone.0179472)
Supplement: S5 Table — (DOCX) [file pone.0179472.s007.docx]

**S5 Table. Numbers needed to harm for BZD- and Z-drug associated pneumonia risk in**

**CKD patients**

|  | **Adjusted OR**  **(95% CI)** | **Number need to harm**  **(95% CI)** |
| --- | --- | --- |
| **BZD** |  |  |
| Current use (0-30 days)^a^ | 1.31 (1.18-1.46) | 478 (322-823) |
| New use ^a^ | 2.47 (2.02-3.03) | 101 (73-145) |
| By dose |  |  |
| ≦0.50 DDD | 1.25 (1.09-1.43) | 593 (345-1,647) |
| 0.51-1.00 DDD | 1.28 (1.09-1.50) | 530 (297-1,647) |
| >1.00 DDD | 1.38 (1.13-1.69) | 390 (215-1,141) |
| By duration |  |  |
| 1-30 days | 1.65 (1.44-1.89) | 228 (167-337) |
| By route |  |  |
| Oral | 1.23 (1.10-1.37) | 645 (401-1,483) |
| Parenteral | 2.88 (1.87-4.42) | 79 (43-170) |
| By individual drug |  |  |
| Chlordiazepoxide | 1.55 (1.22-1.96) | 270 (154-674) |
| Diazepam | 1.55 (1.14-2.12) | 270 (132-1,059) |
| Flunitrazepam | 2.29 (1.26-4.18) | 115 (47-570) |
| Lorazepam | 1.28 (1.03-1.60) | 530 (247-4,942) |
| Midazolam | 2.43 (1.53-3.86) | 104 (52-280) |
| Nordazepam | 3.69 (1.16-11.76) | 55 (14-927) |
| **Z-drug** |  |  |
| New use ^a^ | 2.94 (1.65-5.26) | 76 (35-228) |
| By duration |  |  |
| 1-30 days | 1.75 (1.13-2.72) | 198 (86-1,141) |
| **BZD plus Z-drug** |  |  |
| New use ^a^ | 2.47 (1.10-5.51) | 101 (33-1,483) |
| By route |  |  |
| Oral & Parenteral | 1.93 (1.12-3.35) | 159 (63-1,236) |
| ^a^Recency of BZRAs treatment was classified based on the start date of the most recent BARAs prescription before the index date. New users were defined as patients with any BZRA prescription that started within 30 days, but did not have any record of BARAs in the 31 to 365 days preceding the index date; the remaining current users were defined as continuing users.  Abbreviations: BZD, benzodiazepine; BZRAs, benzodiazepine receptor agonists; CKD, chronic kidney disease; CI, confidence interval; OR, odds ratio. | | |
