# Supplementary material for: Benzodiazepine and Z-drug use and risk of pneumonia in patients with chronic kidney disease: A population-based nested case-control study
Source: PLoS One. 2017 Jul 10;12(7):e0179472. doi: 10.1371/journal.pone.0179472 (PMC5503235; doi:10.1371/journal.pone.0179472)
Supplement: S6 Table — (DOCX) [file pone.0179472.s008.docx]

**S6 Table. Pharmacokinetic parameters and characteristics of analyzed individual benzodiazepine receptor agonists (BZRAs)**

| **BZARs** | **Adjusted OR**^a^  **(95%CI)** | **Active metabolite (s)** | **Half-life**^b^ | **Half-life (hours)^1-4^** | **Route** | **Propylene glycol**^c^ | **Primary**  **Elimination^4,5^** |
| --- | --- | --- | --- | --- | --- | --- | --- |
| Alprazolam | 1.09 (0.84-1.42) | No | Medium | 10-20 | Oral | No | Renal: 80% |
| Bromazepam | 0.83 (0.47-1.46) | No | Medium | 20 | Oral | No | Renal: 70% |
| Chlordiazepoxide | 1.55 (1.22-1.96)^d^ | Yes | Long | 4-100 | Oral | No | Renal: 60% |
| Clonazepam | 0.99 (0.73-1.34) | No | Long | 20-60 | Oral | No | Renal: 49-69% |
| Diazepam | 1.55 (1.14-2.12)^d^ | Yes | Long | 14-200 | Oral, IV | Yes | Renal:75% |
| Estazolam | 1.30 (0.97-1.75) | No | Medium | 17 | Oral | No | Renal: 87% |
| Fludiazepam | 1.13 (0.82-1.56) | NA | Medium | 23 ^3^ | Oral | No | NA |
| Flunitrazepam | 2.29 (1.26-4.18)^d^ | Yes | Long | 16-35 | Oral, IV | Yes | Renal: 87% |
| Lorazepam | 1.28 (1.03-1.60)^d^ | No | Medium | 10-20 | Oral, IV | Yes | Renal: 39% |
| Midazolam | 2.43 (1.53-3.86)^d^ | Yes | Short | 1.5-2.5 | Oral, IV | No | Renal: 45-57% |
| Nordazepam | 3.69 (1.16-11.76)^d^ | Yes | Long | 30-150 | Oral | No | Renal |
| Oxazolam | 1.02 (0.66-1.57) | NA | Short | 4.6 | Oral | No | Renal:50-80% |
| Triazolam | 1.69 (0.72-3.96) | No | Short | 1.5-5.5 | Oral | No | Renal: 80% |
| Zolpidem | 0.95 (0.69-1.32) | No | Short | 0.7-3.5 | Oral | No | Renal: 48-67% |
| Zopiclone | 1.63 (0.86-3.09) | No | Short | 5 | Oral | No | Renal: 84% |
| ^a^The findings from Table 4.  ^b^Half-life is defined as follows: short: < 10 h; medium: 10–24 h; Half-life intervals that exceeded 24 h were considered as long-acting drugs.  ^c^Whether or not parenteral formulations contain propylene glycol.  ^d^*P*-value <0.05.  Abbreviations: IV, intravenously; NA, not available. | | | | | | | |

**References**

1. de Souto Barreto P, Lapeyre-Mestre M, Cestac P, Vellas B, Rolland Y. Effects of a geriatric intervention aiming to improve quality care in nursing homes on benzodiazepine use and discontinuation. *Br J Clin Pharmacol.* 20160321 2016;81(4):759-767.

2. S Louvet, M Ischayek, R Danoff. The Current Role of Long-Term Benzodiazepines for the Treatment of Generalized Anxiety. *Osteopathic Family Physician* 2015;1:19-25.

3. Dainippon Sumitomo Pharma Co. L. Product Information: Erispan-S oral tablets, fludiazepam oral tablets. 2015.

4. Shindo H, Nakajima E, Yasumura A, Murata H, Hiraoka T. Studies on the metabolism of oxazolam. I. Distribution and excretion studies. *Chem Pharm Bull (Tokyo).* 19710403 DCOM- 19710403 1971;19(1):60-71.

5. Lemaire-Hurtel, Anne-Sophie, Alvarez J-C. Drugs Involved in Drug-Facilitated Crime—Pharmacological Aspects. *Kintz P, ed. Toxicological Aspects of Drug-Facilitated Crimes*. 1 ed: Academic Press(Elsevier); 2014:53-62.
