# Supplementary material for: Benzodiazepine and Z-drug use and risk of pneumonia in patients with chronic kidney disease: A population-based nested case-control study
Source: PLoS One. 2017 Jul 10;12(7):e0179472. doi: 10.1371/journal.pone.0179472 (PMC5503235; doi:10.1371/journal.pone.0179472)
Supplement: S2 Fig — (DOCX) [file pone.0179472.s010.docx]

**S2 Fig. Flow Diagram Depicting Selection Process of Study Subjects.**
